# Recent technologies in cardiac imaging

**DOI:** 10.3389/fmedt.2022.984492

**Published:** 2023-01-10

**Authors:** Quinn Counseller, Yasser Aboelkassem

**Affiliations:** ^1^ College of Health Sciences, University of Michigan, Flint, MI, United States; ^2^ College of Innovation and Technology, University of Michigan, Flint, MI, United States; ^3^ Michigan Institute for Data Science, University of Michigan, Ann Arbor, MI, United States

**Keywords:** cardiac imaging technologies, MRI, CT, multimodal, machine learning, artificial technology

## Abstract

Cardiac imaging allows physicians to view the structure and function of the heart to detect various heart abnormalities, ranging from inefficiencies in contraction, regulation of volumetric input and output of blood, deficits in valve function and structure, accumulation of plaque in arteries, and more. Commonly used cardiovascular imaging techniques include x-ray, computed tomography (CT), magnetic resonance imaging (MRI), echocardiogram, and positron emission tomography (PET)/single-photon emission computed tomography (SPECT). More recently, even more tools are at our disposal for investigating the heart’s physiology, performance, structure, and function due to technological advancements. This review study summarizes cardiac imaging techniques with a particular interest in MRI and CT, noting each tool’s origin, benefits, downfalls, clinical application, and advancement of cardiac imaging in the near future.

## 1. Introduction

Cardiovascular disease is a substantial factor in premature death and disability worldwide (1). Diagnostic imaging may positively influence overall population health, morbidity, and quality of life through early and effective means. The goal is to utilize cardiovascular imaging as a preventative measure rather than a reactive strategy to reduce cardiovascular issues by catching concerns early. However, the strengths and limitations and the array of imaging options may be an obstacle for the clinician (2). From the earliest development, standard imaging techniques are as follows: echocardiogram via ultrasound, x-ray, computed tomography (CT), nuclear scans, magnetic resonance imaging (MRI), and catheterization. The scanning technologies and associated ionizing radiation, typically measured in millisieverts (mSv), are illustrated in Table 1.

**Table 1:** Scanning technologies and associated ionizing radiation measured in millisieverts (mSv).

Test	Radiation exposure (mSv)
Echocardiogram	0.0
MRI	0.0
Chest x-ray	0.05
Calcium scoring test	1–2
Cardiac catheterization	7
Chest CT	10
Coronary CT angiography	3–14
Radionuclide stress test	10–12
Radionuclide myocardial perfusion imaging	25

**Figure 1:**
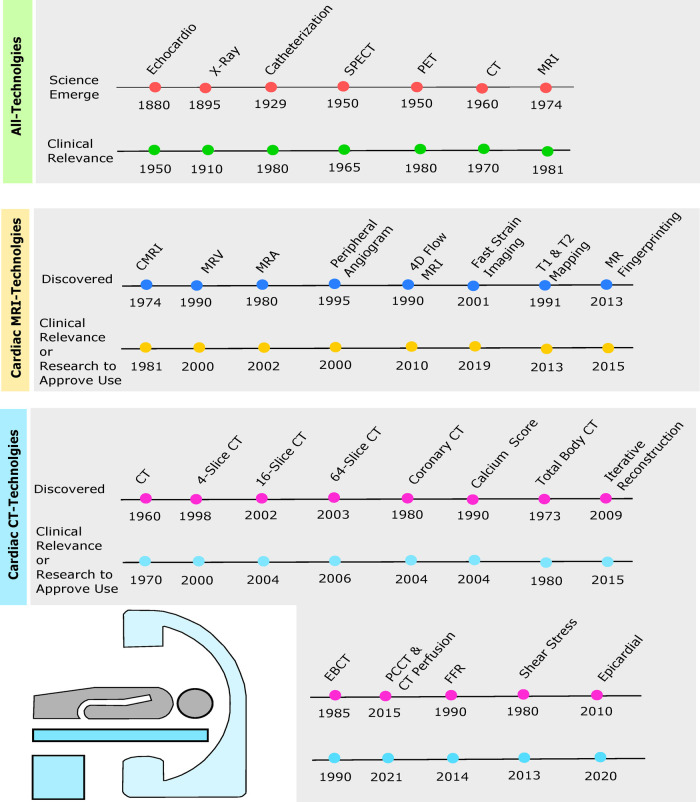
Timelines depict originating year of cardiac imaging invention followed by the year of clinical relevance or enough evidence from research to be approved by the Food and Drug Administration (FDA) for clinical use. Green timeline includes echocardiogram, chest x-ray, cardiac catheterization, SPECT, PET, cardiac CT, and cardiac MRI. Yellow timeline depicts cardiac MRI, magnetic resonance venography (MRV), magnetic resonance angiography (MRA), peripheral MRA, 4D flow, fast strain-encoded (fast-SENC) cardiac imaging, T1 and T2 mapping, and cardiac magnetic resonance (MR) fingerprinting (MRF). Blue timeline shows cardiac CT, 4-slice CT, 16-slice CT, 64-slice CT, coronary CT, calcium score, total body CT, iterative reconstruction (IR), electron beam CT (EBCT), photon-counting CT (PCCT), fractional flow reserve (FFR), wall shear stress (WSS), and epicardial fat enhancement technology.

The idea of imaging technology has been around since the 1880s, but often, these technologies would advance and translate to evaluate the heart several decades later (3). For example, the echocardiogram (echo) was developed in 1880 but was not clinically relevant until the 1950s, see Figure 1. Echocardiography was created as a diagnostic test to examine cardiac function, structure, and hemodynamics (4). It uses ultrasound, also known as sonography, to produce high-frequency sound waves which bounce from the heart to the transducer to create a visual of the heart on the computer. Various types of echos are available to examine the heart, such as transthoracic echo (TTE), three-dimensional (3D) echo, intracardiac echo (ICE), M-mode echo, transesophageal echo (TEE), Doppler echo, and stress echo, each having unique benefits (5). Overall, the advantages of an echo are a more detailed picture than an x-ray and no exposure to radiation. The moving graphic also gives additional information on the pumping of the heart’s chambers and the structure of the walls. Abdominal/thoracic aortic aneurysm, blood clots, pericarditis, pericardial effusion, and valvular heart disease are just a few examples of what an echo typically screens for (6).

**Figure 2:**
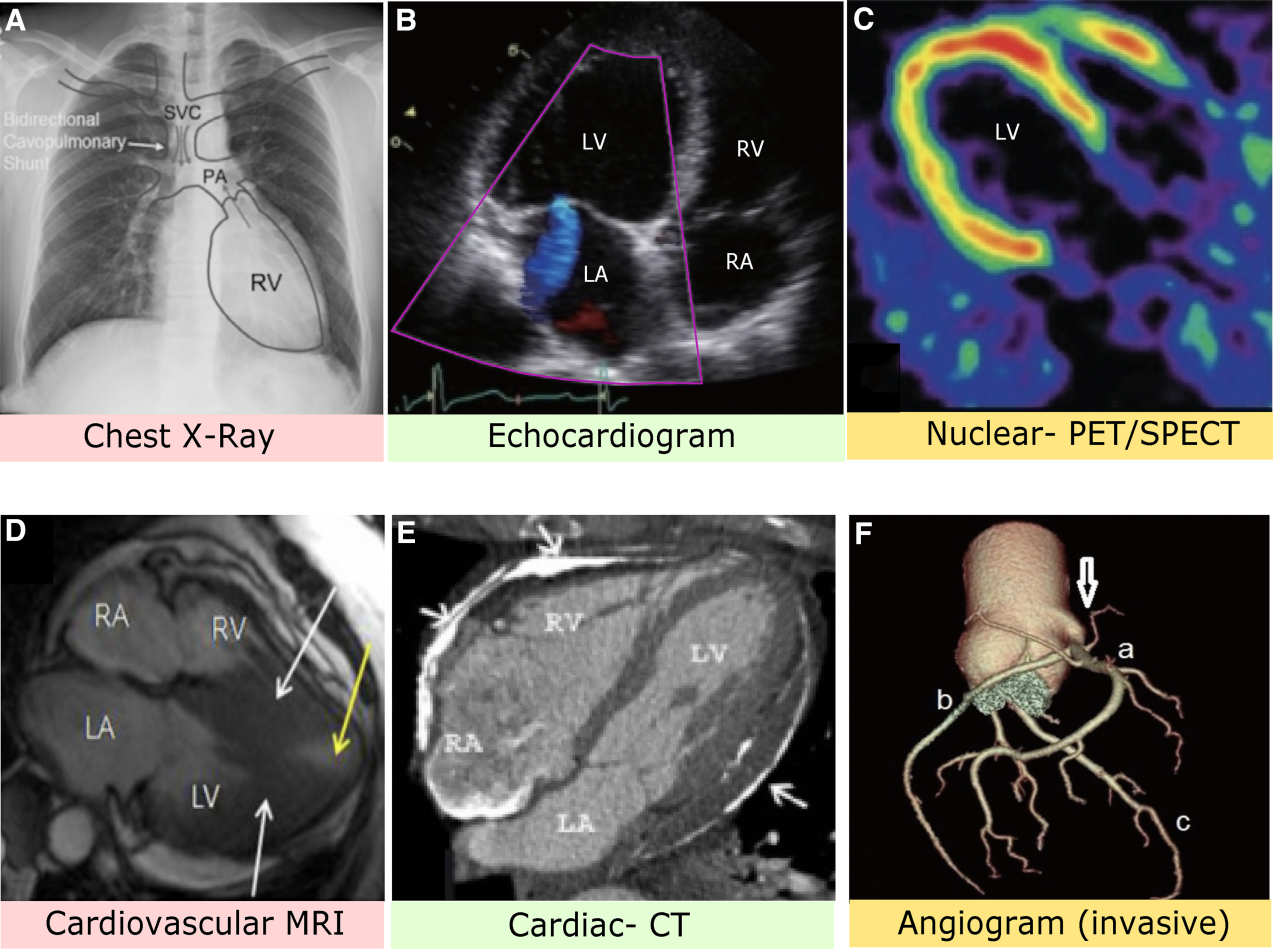
(**A**) Chest x-ray image with anatomical identification of relevant heart structures. Grey arrows represent blood flow. SVC, superior vena cava; PA, pulmonary artery; RV, right ventricle (8). (**B**) Stress-induced cardiomyopathy via echocardiogram depicting apical four heart chamber view. LV, left ventricle; LA, left atrium; RV, right ventricle; RA, right atrium (9). (**C**) PET imaging with contrast allows assessment of blood flow and cardiac sympathetic innervation of the LV (10). (**D**) MRI with nuclear enhancement revealed an obstructed LV (white arrows) and decreased blood flow. The patient had complaints of angina and dyspnea (11). (**E**) Axial CT image identifies extensive plaque of pericardial layers (arrows) and deformity suggestive of pericardial constriction (12). (**F**) Computed CT angiogram indicates the right coronary sinus (arrow) with a mitral valve anomaly. A: Right coronary artery, B: Left circumflex artery, C: Left anterior descending artery (13).

A heart x-ray, commonly called a chest x-ray (CXR), creates a visual of the heart, lungs, and surrounding bones via radiation beams and was developed in 1895 (7). Chest radiographs are primarily used to detect the anatomy of the aorta, pulmonary veins, and pulmonary arteries. Regarding producing a visual, the body varies in depth and thickness of tissue structures; thus, amounts of radiation are absorbed differently, see Figure 2. For example, soft tissues (e.g., blood, fat, skin, muscle) may look dark grey while bones may look white (14). CXRs are typically used when patients come in due to dyspnea, persistent coughs, or angina. It is most commonly used because the scan is prompt, straightforward, relatively affordable, and generalizable, meaning it can be used to screen a vast number of possible conditions responsible for symptoms.

The first CT scanner was invented in the 1960s and clinically applied in the early 1970s (15). It merges a sequence of cross-sectional slices of the heart and surrounding blood vessels via x-ray images. A significant difference to CXR is cardiac CT can create 3D illustrations. Cardiac CT aims to provide highly detailed images of blood vessels, soft tissues, and bones in less than 20 minutes. As for types of CT scans, a coronary artery calcium (CAC) scan detects the presence and proportions of plaque in arteries (16). A coronary CT angiogram (CTA) quantifies the amount of calcified and non-calcified plaque, indicating the severity of stenosis (17). Cardiac CTs are generally ordered for angina, atherosclerosis, coronary artery disease (CAD), dyslipidemia, and obstructed coronary artery disease.

In the 1960s, the single-photon emission computed tomography (SPECT) scan was developed closely alongside CT and became clinically relevant in the 1970s (18). Before a SPECT scan, a radiolabeled tracer that emits gamma rays is injected into an individual’s bloodstream. This collects information from the gamma rays and displays visuals of the heart on the CT cross-sections to rule out perfusion, scar tissue, or see if bypass or other cardiac surgery procedures are working as they should. One type of particular test is called the cardiac SPECT perfusion, typically performed using a cardiac stress test to examine the functional status of the heart at rest and under activity or stress (19). A physician may order a SPECT if a patient has symptoms of heart disease, assess the risk of myocardial infarction, or investigate damaged cardiac muscle via examining blood flow.

**Figure 3:**
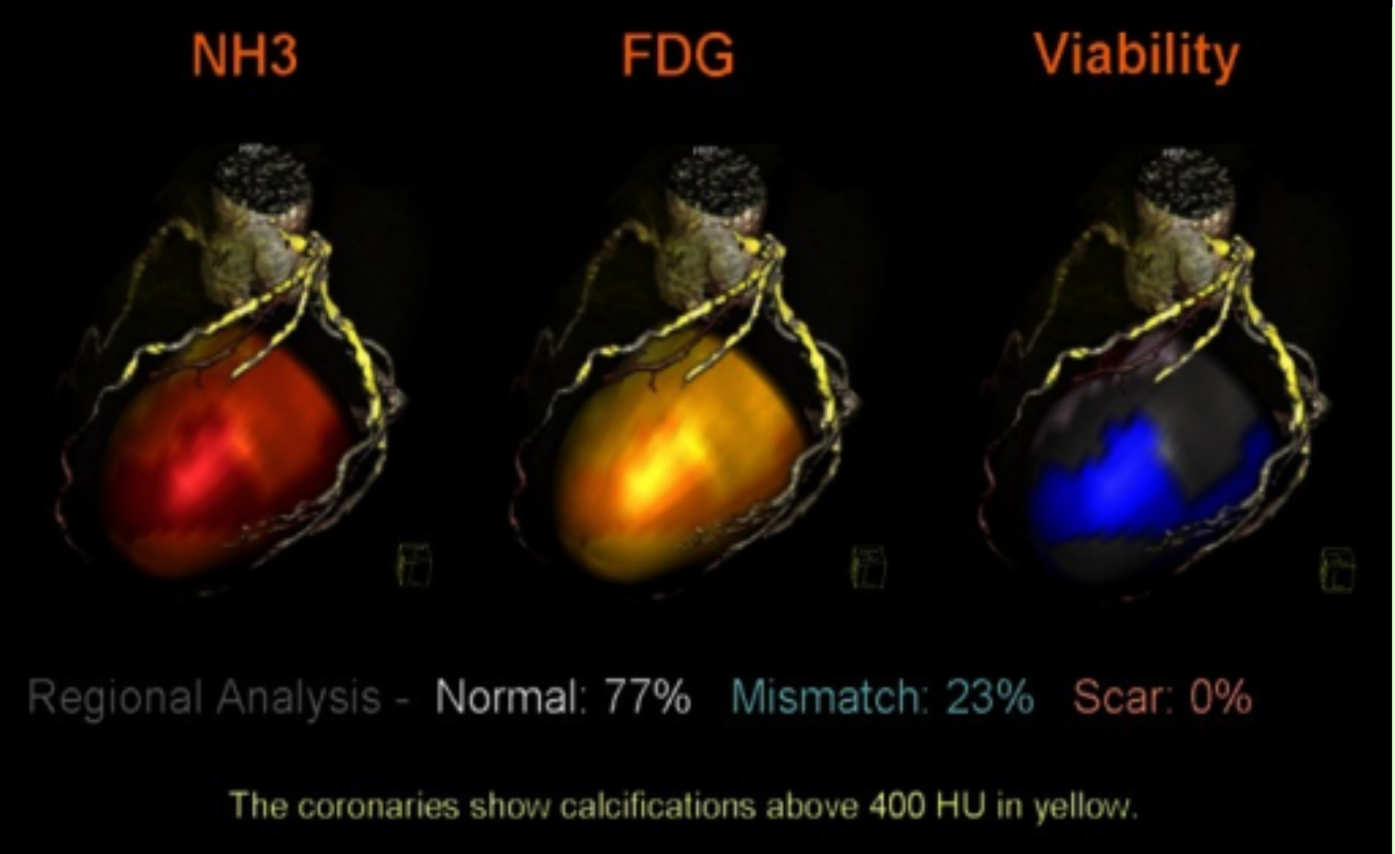
PET perfusion data mapped onto the epicardial surface of CT angiography to evaluate the viability of cardiac muscle and segmented coronary tree. The image depicts the simultaneous mapping of metabolism, coronary anatomy, and myocardial perfusion via fused images. NH3, ammonia, FDG, F-fluorodeoxyglucose, HU, Hounsefield units (23).

Shortly after SPECT was developed, a positron emission tomography (PET) scan followed (20). Similarly, PET is a nuclear and molecular imaging technique that injects a small radioactive tracer to circulate through the heart. The difference between SPECT and PET is the type of radiotracer used; SPECT measures gamma rays, whereas PET scans produce positrons (21). As for the process of PET scan, the radiotracer is injected, the computer detects the radiation from the tracer to determine damage to tissue, viability, and abnormal substance buildup, which then produces an image. A particular type of test commonly used is called cardiac PET viability, used to evaluate whether heart cells are healthy and fully functional (22). Both SPECT and PET scans are beneficial for the high level of detailed information they provide on blood flow. They can diagnose a person who may have symptoms of heart disease or determine the amount of damage for an individual with a history of myocardial infarction. They are particularly useful in the progression of cardiac diseases, such as characterizing occluded coronary arteries. See Figure 3 below that illustrates PET perfusion data mapped onto the epicardial surface of the heart.

In 1981, the first magnetic resonance imaging (MRI) visuals of the human heart were obtained (24). However, MRI quality was sub-par compared to others at this time. Over the following decades, hardware improvements and dual-inversion techniques contributed to unmatched imaging capabilities. MRI enables noninvasive imaging through magnetic fields that propel the body’s hydrogen atoms to align temporarily. Radiofrequency is then pulsed, stimulating the protons out of equilibrium from straining against the force of the magnetic field. MRI fiber optic temperature sensors detect the energy and depict cross-sectional MRI images (25). The unit of measurement used to quantify the power of the magnetic field is called a Tesla (T), typically ranging from 1.5 to 3 T (26). The differences in proton activity allow the MRI sensors to detect the energy, which translates to images on the screen that the radiologist can review and interpret. Distinct types of scans include cardiac viability MRI, right and left ventricle function MRI, stress perfusion MRI, structural assessment MRI, and MRI angiogram. An MRI scan may help examine the heart’s function, structure, beating pattern, blockage of arteries, or damage to the heart muscle. A significant advantage compared to other scans is the lack of radiation and strenuous iodine contrast.

Catheterization was discovered in 1929, but cardiac catheterization, also known as cardiac angiography, did not become clinically relevant until the 1980s (27,28). Cardiac catheterization, a type of invasive cardiac imaging, works by guiding a thin, flexible tube, called a catheter, into a blood vessel, usually the femoral vein, to the heart to diagnose or treat heart conditions. It allows physicians to look for stenosis, irregular arrhythmias, and gain important information about the general functioning of the heart muscle, valves, and vessels. Types of cardiac catheterization include coronary angiogram or angiography, fractional flow reserve (FFR), intravascular ultrasound (IVUS), optical coherence tomography (OCT), and vascular function testing, including endothelial function testing and index of microcirculatory resistance (29). Cardiac catheterization may be used for treatment involving angioplasty, stenting, heart valve repair/replacement, and diagnostic testing. Opposed to other imaging techniques, catheterization is an invasive procedure. Additionally, it may come with coronary-intervention related complications such as hematoma or retroperitoneal bleeding, catheter-induced dissection (e.g., vascular dissection), arrhythmias, kidney damage, and more (30).

The novelty of this study is a comprehensive review summarizing cardiovascular imaging technology to bridge the technical and clinical aspects for a more holistic and practical approach to medicine. The second aim of this study is to investigate gold standards for each cardiac imaging technology in diagnosing cardiovascular conditions, particularly MRI and CT. Thirdly, we explore more recent and innovative imaging techniques relevant to diagnosing and correcting cardiovascular issues and what we can expect to see for cardiovascular imaging in the future.

## 2. Cardiac MRI

MRI is referred to as the gold standard due to its precision, reliability, and specificity (31). MRI can be used in myriad ways, such as risk stratification, noninvasive volumetric and functionality assessment of ventricles, assessment of myocardial viability, tissue characterization, size/function of heart’s chambers, and thickness of movement of the walls of the heart (32). It can also screen for the extent of damage caused by heart disease or heart attacks, identify structural problems in the aorta (e.g., aneurysms or dissections), examine stress function (e.g., dobutamine or exercise), quantify blood flow, and identify inflammation or blockages in blood vessels (33,34). MRI is most frequently used to image the central nervous system, including the brain and the spinal cord. For cardiac MRIs, they have proven to be the most valuable in detecting cardiovascular anatomical abnormalities, functional anomalies, conditions related to CAD and cardiomyopathy, and finding tumors.

### 2.1. MRI types

Types of MRI include magnetic resonance angiography (MRA), magnetic resonance venography (MRV), cardiac MRI (cardiac viability, right and left ventricle function, structure, perfusion), and peripheral MRA (35). Each has unique purposes and benefits, see Table 2. These imaging techniques use gadolinium contrast medium, sometimes called contrast agents, dyes, or media. When injected into the body, the gadolinium agent enhances the quality of MRI images. This allows the radiologist to interpret or detect abnormalities more accurately.

**Table 2:** Types of MRI scanning technologies and the identified medical condition.

Type	Identified medical condition
MRA:	Abnormalities in arteries, detect atherosclerotic disease
	Identify arteriovenous malformation
MRV:	Abnormalities in veins and detect blood clots
Cardiac MRI:	Detect or monitor cardiac disease, evaluate heart anatomy and evaluate heart function
	Examine blood flow, evaluate effects of coronary artery disease
	Planning or monitoring treatment
Peripheral MR angiography:	Evaluating peripheral vascular disease and identifies plaque in arteries

### 2.2. Design

Regarding design, MRIs vary in weight, building vibration, sound interference, bore size, and magnet field strength. Clinical MRIs typically weigh from 11,000–17,600 pounds (e.g., 5,000–8,000 kg), but may vary depending on make and model (36). The significant weight is for two reasons: the numerous heavy magnets and the cooling system to maintain the magnet stability. As for building vibration, robust vibrations due to Lorentz’s force produced by fast switching currents from gradient coils within the MRI scanners often create undesirable acoustic noises (37). This sound interference, also known as MRI knocking, depends on the sequence used. MRI acoustics have proposed solutions to this rhythmic knocking sound, including noise-canceling headphones or earplugs for the patient, acoustic reduction technology, and silent scanning technology (38). Next, onto the three bore sizes. There is a closed bore, wide bore, and open MRI. The standard closed bore provides high-quality and high-clarity images, whereas the open bore tends to have less precision with visual outputs and weaker magnets. A closed bore MRI is typically 60 centimeters wide vs. 70 cm with a wide bore. Additionally, the wide bore can support up to 550 pounds, giving it a more significant weight limit than standard scanners (39). An open MRI is unique in style, with two flat magnets stacked on top of each other with a space in between for the patient to lay in. The MRI technician would schedule this scan for a patient with extreme claustrophobia. Clarity is diminished similarly to the wide bore MRI. Magnet field strength, to date, has varied between 0.5–11.7 T in pre-clinical studies and in clinical MRIs 0.5–3 T is preferred, but it is typical to expect a 1.5 or 3 T MRI scanner (40). As for research studies, benefits and risks are being examined for higher strength magnets to quicken scanning times with 7–11.7 T levels.

### 2.3. Disease-specific protocols

Cardiac MRI has general techniques for formulating images of the heart in areas of (a) left ventricular structure and function, (b) right ventricular structure and function, (c) first-pass perfusion, (d) late gadolinium enhancement, (e) stress perfusion, (f) stress function, (g) blood flow quantification, (h) advanced tissue characterization (T1 and T2 mapping), and (i) rapid protocols (41). More specifically, these MRI techniques can find specific diseases. For example, ischemic heart disease, and within that particular myocardial injury, whether it is an acute or chronic disease, edema, necrotic injury, wall thickening, perfusion, and viability. See Table 3 for extensive list of extensive range of cardiovascular diagnoses and conditions that can be found with MRI.

**Table 3:** Medical condition and diagnosis that can be identified by using cardiovascular MRI scanning.

Imaging type	Diagnosis and condition
Cardiovascular MRI	Abdominal aortic aneurysm and arrhythmogenic ventricular cardiomyopathy (AVC)
	Atherosclerosis and atrial fibrillation (A-fib)
	Atrial flutter, atrial tachycardia and atrioventricular nodal reentrant tachycardia (AVNRT)
	Bradycardia and cancer-related cardiomyopathies
	Cardiac and paracardiac masses, including thrombi and cardiac tumor
	Cardiac sarcoidosis and congenital heart disease
	Cardiomyopathy, chronic ischemic heart disease and viability
	Coronary artery disease and coronary artery evaluation
	Dilated cardiomyopathy
	Heart attack/acute MI/acute coronary syndromes
	Heart failure, hypertension heart disease and hypertrophic cardiomyopathy (HCM)
	Left ventricular non-compaction
	Myocarditis
	Paroxysmal supraventricular tachycardia (PSVT)
	Pericarditis/pericardial disease and post-heart transplantation
	Pulmonary vein evaluation/pre- and post-ablation
	Recreational drug-induced cardiomyopathies and restrictive cardiomyopathy
	Siderotic cardiomyopathy and spontaneous cardiac artery dissection (SCAD)
	Thoracic aortic aneurysm
	Valvular heart disease (specific approaches by valve-mitral, aortic, tricuspid pulmonic)
	Ventricular fibrillation (V-fib)

### 2.4. Physics and imaging sequences

**Figure 4:**
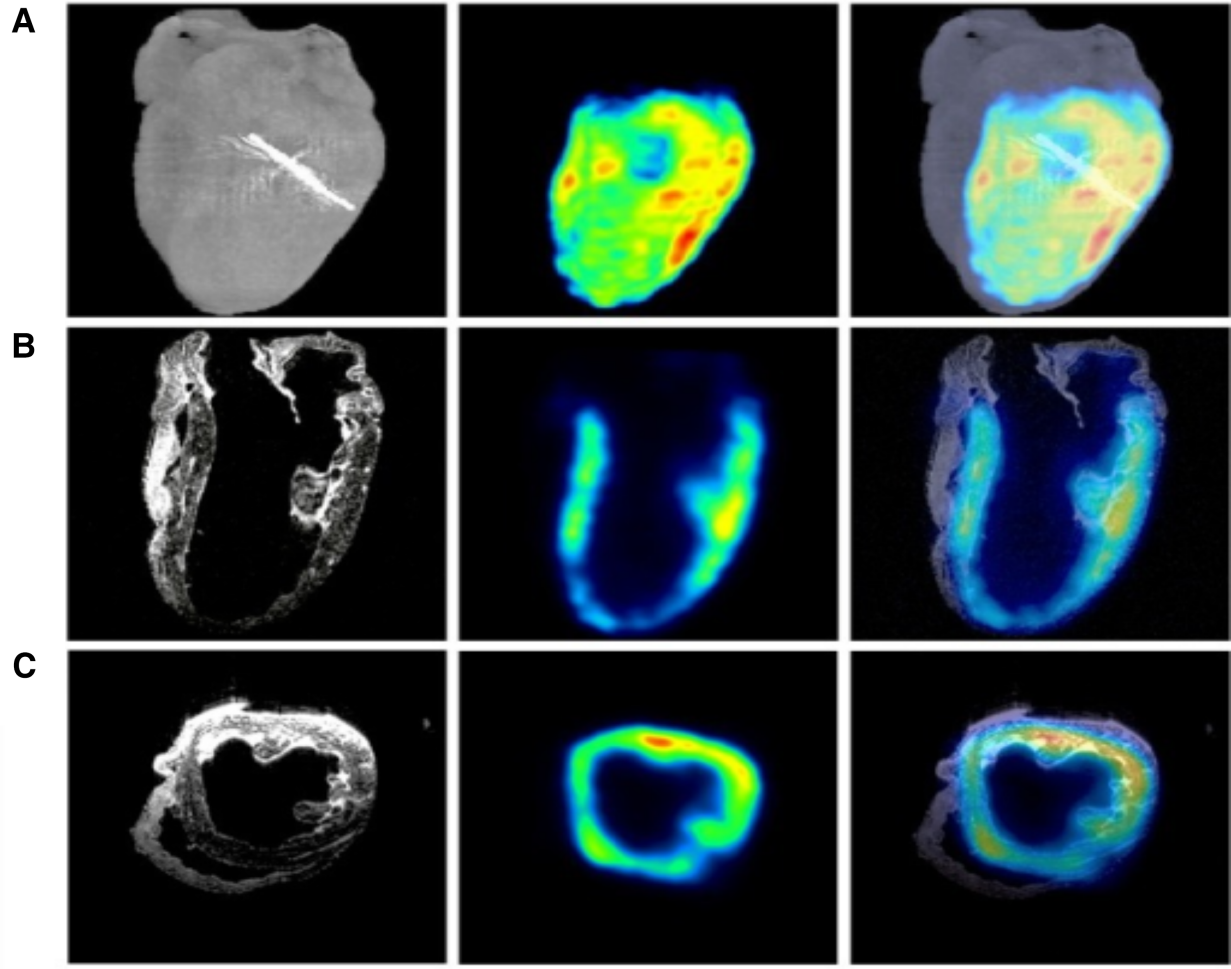
MRI, PET, and micro-CT are utilized to image a regionally ischemic heart. (**A**) Volumetric rendings with micro-CT and PET are merged acquisitions to indicate the site of left anterior descending coronary artery ligation. (**B**) Coronary slices from MRI and PET scans combined with the merger of another MRI and PET scan images are depicted. (**C**) Transverse slices from MRI, PET, and the merged images form an analogous perfused heart. Hypointense T2-weighted MRI signals from ferrum (iron) couple with PET signals (45).

MRI is based on the concept a uniform external magnetic field uses radiofrequency energy to align protons in the human body. Fourier transformation is used to relate the frequency information in each imaged plane to corresponding intensity grades, arranged in a matrix of pixels and depicted in shades of grey (42). Tissue is then characterized by longitudinal relaxation time (T1) and transverse relaxation time (T2), defined as the time taken for spinning protons to realign with the magnetic field and the time taken for spinning protons to reach equilibrium; respectively (43). The most commonly used clinical MRI sequences are T1- and T2-weighted scans. T1-weighted uses a short repetition time (successive pulse sequences) and a short time to echo (delivery of radiofrequency pulse and receipt of the echo signal). T2-weighted uses a long repetition time and a long time to echo (44). See Figure 4 below that illustrates enhancements of iron with the help of T2-weighted MRI signals. Another commonly used imaging sequence is fluid-attenuated inversion recovery (FLAIR). It is similar to T2, with a long repetition time and a long time to echo. But in this case, the times are even longer than T2. In doing so, abnormalities are more luminous and more sensitive to pathologies than T1 and T2 sequences (46). Diffusion-weighted imaging (DWI) detects spontaneous movements of water protons (47). In the extracellular space, molecules diffuse willingly, whereas they are significantly confined in the intracellular space. Diffusion becomes restricted considerably in areas of cardiac ischemia, the sodium-potassium pump shuts down, and sodium accumulates intracellularly, thus forming an osmotic gradient. Lack of spontaneous movement intracellularly creates a bright signal on DWI, a favorably sensitive method for detecting ischemia, infarction, acute myocarditis, and more (48).

### 2.5. MRI future

#### 2.5.1. Efficiency with 4D flow

A significant focus has been on shortening scan time to perform cardiac MRIs more efficiently. For example, in-plane phase-contrast imaging provides a time-resolved cine sequence that allows three-dimensional velocity encoding, a four-dimensional (4D) flow technique (49). The 4D flow is a type of phase-contrast that uses the movement of blood encoded in three different orientations, resolved to three spatial and temporal dimensions in cardiac circulation (50). In other words, 4D flow can analyze a vessel at any level. Detection of hemodynamic principles includes shear stress, turbulent kinetic energy, pressure gradient, flow components, and pulse wave velocity. Although the high spatial resolution and extensive volume coverage add to MRI 4D flow usefulness, downsides include long scan time and post-processing time. However, new studies show accelerated scan times, some as brief as two minutes to examine aortic blood flow (51). Additionally, other studies have found that 4D flow sequencing not only shortens imaging time but has improved dataset pre- and post-processing (31). Azarine and colleagues (49) provided examples of vendors and post-processing software programs already in use for cardiac segmentation to improve the speed and accuracy of the correction process. They predict increasing outputs with less processing time as the segmentation process develops with machine learning and deep learning. As for MRI efficiency and shortening scan times, other studies have focused on altering clinical magnet strength from 1.5 to 3.0 T for more accurate images, eliminating motion artifacts by increasing the speed and overall quicker scan times. However, others continue to recommend 1.5 T as they find less noise and artifact with images of the myocardium (52).

#### 2.5.2. Fast strain-encoded imaging

Myocardial strain imaging uses cardiac ultrasound to evaluate the myocardium’s function or deformation (53). It is a non-invasive method used to identify subtle changes in functioning related to the four chambers of the heart and blood flow. Strain imaging combined with cardiac MRI and echo can be utilized through different imaging processing algorithms such as tissue Doppler imaging, speckle tracking echocardiography (STE), MRI tagging, and MRI feature tracking (54). Echo is primarily responsible for Doppler imaging, examining strain rate in a pulsed wave formation due to cardiac blood flow and STE, where spatial translocation of speckles, also known as derived functional units, allows quantification of heart function (55). In contrast, MRI is mainly responsible for tagging and feature tracking. Strain analysis may be used by acquiring specific targeted sequences, also known as tagging, or post-processing techniques on standard cine sequences such as feature tracking. Fast strain-encoded (SENC) cardiac MRI imaging (fast-SENC) is a newer technique that captures single cardiac contractility in a single heartbeat (56). However, due to additional acquisition time, acquisition sequence required, and time-consuming post-processing algorithms, MRI tagging today is mainly used for research (57).

#### 2.5.3. T1 and T2 mapping with ECV

Parametric T1 and T2 mapping paired with extracellular volume fraction (ECV) quantification can provide quantitative measurements of the myocardium muscle and associated edema (58). Kim and colleagues (44) found the interaction between T1, T2, and ECV values and associated findings help rule out or determine dilated cardiomyopathy, hypertrophic cardiomyopathy, Fabry disease, myocarditis, amyloidosis, ischemic cardiomyopathies (e.g., acute or chronic MI), or other cardiomyopathies (e.g., systemic lupus erythematosus, systemic sclerosis, cardiac iron). Another study experimented with a three-parameter model incorporating concurrent T1 and T2 mapping named multiparametric saturation-recovery single-shot acquisition (mSASHA) during a single breath-hold (59). They found high accuracy with quantification. One study investigated a similar technique to mSASHA, called a modified look-locker (MOLLI), using T1 sequencing. They discovered that MOLLI is less accurate but more precise than mSASHA. Therefore, they combined the MOLLI and mSASHA sequences to create an overlapping technique for synthetic ECV measurements but ultimately found similar findings (60). It is transparent that T1 and T2 mapping are gaining traction to detect cardiomyopathies and predictive characteristics for monitoring and prognosis. Although some models remain in the research stage, they are expected to be translated into clinical use shortly in the future.

#### 2.5.4. Cardiac MR fingerprinting

A technique called cardiac magnetic resonance (MR) fingerprinting (MRF) is another quantitative parametric tool that quantifies T1 and T2 values during a breath-hold (61). It is similar to T1 and T2 mapping with ECV but ties in another component. In addition to the late gadolinium enhancement, it quantifies tissue-specific parameters such as T1, T2, T2* relaxation times, and ECV to characterize and detect immediate and diffuse myocardial illness (62). MRF provides co-registered, simultaneous, and multiparametric mapping during a single scan, and initial studies have found MRF achieves comparable map quality to conventional methods in reduced scan times. However, biases have been detected due to sequences (e.g., MOLLI, SASHA), vendors, and confounding factors (63). Researchers have been working to reduce this bias to achieve a truly quantitative cardiac MRI tool. Potentially, MRF could contribute to this objective due to its flexibility to integrate model modifications, reducing confounding factors (64). Clinical validation of cardiac MRF is in the extremely early stage, but there have been encouraging results with heart transplant patients and patients with suspected inflammatory and hypertrophic cardiomyopathies (62). Further validation is required to assess the reproducibility of the technique to replace available mapping procedures.

#### 2.5.5. Multimodality integration

**Figure 5:**
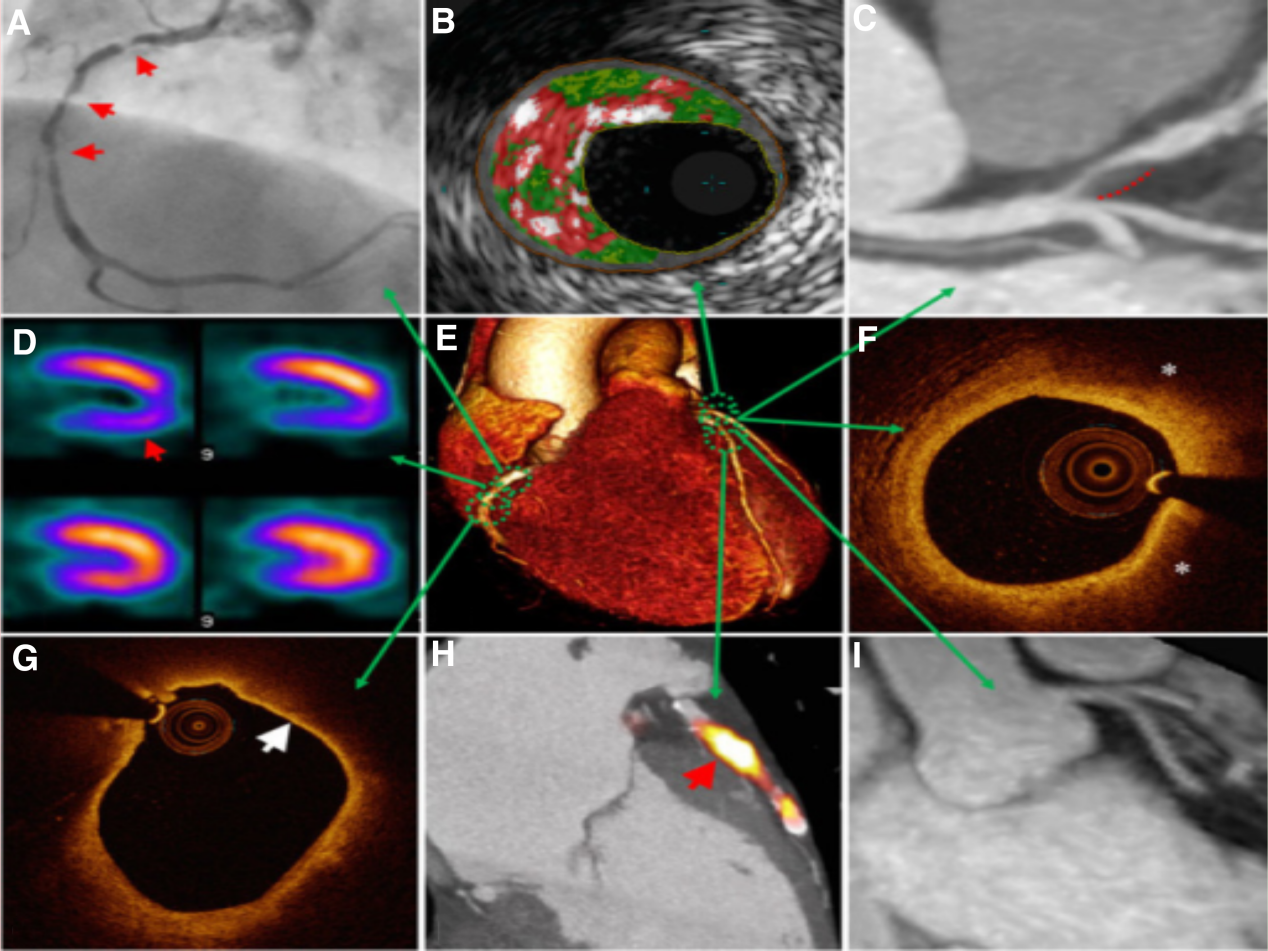
Multimodal technique to atherosclerosis imaging. Each modality offers respective measurements to aid in diagnosis or treatment. Combining the information on anatomic and hemodynamic properties, calcification, and metabolic activity allows accurate analysis of an individual patient. (**A**) X-ray angiography with atherosclerotic lesions (arrows); (**B**) Histology intravascular ultrasound illustrating plaque with elevated content of necrotic core (red), calcium (white), and fibro-fatty regions (dark/light green); (**C**) CT angiography revealing non-calcified plaque in the left anterior descending artery (dashed line); (**D**) SPECT myocardial perfusion scan with stress-induced perfusion defect (arrow); (**E**) 3D CT whole-heart image; (**F**) Optical coherence tomography (OCT) image of calcification showing lipid (*); (**G**) OCT image showing thin overlying fibrous cap (arrow), indicative of thin-cap fibroatheroma; (**H**) PET–CT image displaying active microcalcification; (I) MRI contrast-angiography showing clear delineation of coronary vessels (65).

A multimodal approach means employing other imaging techniques, combining their results, and then using all complementary information as a whole unit to solve problems. See Figure 5 for clear representation of multimodality imaging. One criticism of cardiac MRI is the isolation in presentation, with little clinical and functional data integration (66). For example, A group of researchers (67) have shown that patient positional changes with multimodal approaches may be another promising direction for cardiac MRI. Although ergometers have been around for stress testing to assess the heart functionality at rest and under stress, this test has been designed explicitly for asymptomatic patients with valvular heart disease and heart failure with preserved ejection fraction. In supine, an MRI-compatible exercise ergometer can be arranged, then cine and strain imaging can be acquired while the patient is cycling in a supine position (68). Conclusions included feasibility during maximal intensity exercise with real-time imaging, while limitations included flawed acquisition techniques and lengthy scan length (67,68).

#### 2.5.6. Inclusivity

Immersive entertainment systems are being marketed by MRI companies across the globe, bringing a movie theater-like experience to patients undergoing an MRI (69). New advancements to increase inclusivity include efforts to decrease the fear of claustrophobia. The patient is provided a headset and visor coupled with audiovisual technology to distract their senses while in the scanner and reduce anxiety due to confinement to a small space. This approach has been shown to provide a more enjoyable experience due to the entertainment piece, increased distraction, and decreased anxiety pre-scan and during the scan (70).

New protocols are being developed using a wideband imaging technique to overcome artifact-related limitations, including magnetic hardware in the body such as pacemakers and defibrillators. One study used a 0.55 T magnet and found both pacemakers and defibrillators were safe at this level, and researchers could obtain quality illustrations. By combining low field strength with high-performance scanning technology, one study found the O.55 T magnet outperformed the 1.5 T magnet with MRI-guided catheterizations with metal devices, MRI in high-susceptibility regions, and efficient imaging (26).

## 3. Cardiac CT

Computerized axial tomography (CAT) scan and CT scan are the same type of diagnostic imaging, but the more contemporary terminology is the CT scan. Other common names are rotational x-ray, or when dealing with the heart, cardiac CT or an extensive heart x-ray. In a cardiac CT scan, x-ray beams are rotated around the heart and variably attenuated or absorbed by cardiac anatomical structures in a cardiac CT. For example, calcium in an artery (e.g., plaque) will appear brighter or white than in blood vessels (71). As ‘tomography’ means ‘representation of cross-section’, it takes slices reconstructed from all beams to create 3D images. Comparably, 2D images are x-rays or radiographs. Cardiac CT is known for its speed, reliability, and high resolution (72). CT can assess the extent of coronary stenosis (e.g., catheterization can assess if stenosis is present) and visualize the thoracic aorta. Cardiac CT can be used in various ways, such as aneurysms, assessing for complications with procedures, screening for atherosclerosis, evaluating tumors or blood clots, detecting injury to valves, pericarditis, and preparing for dissection or other procedures (73). CT is most frequently used to evaluate the cause of chest pain and dyspnea.

### 3.1. Types

Types of cardiac CT include coronary CT angiogram (CCTA), calcium-score screening heart scan, and total body CT scan; see Table 4 for more detailed overview (74). Each type of scanning technique has individual objectives and advantages. These imaging techniques use harsh iodine contrast, compared to the preferred gadolinium medium (which is metal-based), as CT contrast can damage the kidneys. One study found gadolinium is associated with a lower incidence of contrast nephropathy and early progression to end-stage renal disease in patients with pre-existing chronic kidney disease (75). However, other studies found that the real-life risk is insignificant by undergoing precautions (76). Nevertheless, iodine contrast is often critical as it enhances the quality of the images and allows the radiologist to detect abnormalities sufficiently. The CCTA identifies plaque and blockages or stenosis of the coronary arteries. The calcium-score screening heart scan, also known as a coronary calcium scan, is very accurate at predicting calcium deposits in the heart’s coronary arteries (77). The total body CT scan can grossly detect a wide range of heart issues, including heart attack and CAD.

**Table 4:** Types of CT scanning technologies and the identified medical condition.

Type	Identified medical condition
Coronary CT angiogram:	Coronary blockage
	Determines if fatty or calcium deposits have built up
	Identify need for intervention (e.g., stent)
	Stenosis in coronary arteries
Calcium-score screening heart scan:	Detect atherosclerotic plaque in coronary arteries
	Evaluate risk for coronary artery disease
	Measures calcium in coronary arteries
Total body CT scan:	Aortic aneurysm
	Calcium deposits within plaque in coronary arteries

### 3.2. Design

CTs vary in weight, size, and radiation exposure as far as design goes. Clinical CTs can wildly differentiate in weight due to the diverse selection on the market. Portable CT scanners can weigh as light as 500 pounds, and clinical stationary CTs may weigh several thousands of pounds (78). Patients are typically more comfortable in a CT scanner than in an MRI due to shorter scans, less noise, and it is not as small or claustrophobic (79). The gantry opening size ranges from 70–80 cm in diameter with weight limits as high as 675 pounds (80). Patients typically lie supine on the scanner, and increased stillness equates to less artifact and higher imaging grade, just as MRI. Increased attenuation may also cause the images to look ‘noisy’ and reduce the diagnostic value of the scan. Radiation dose varies according to the scan, with the calcium scoring test having radiation of 1–2 millisieverts (mSv), chest CT of 10 mSv, and coronary CT angiography of 3–14 mSv, see Table 1 (81). All types of CT are non-invasive. Some individuals may misidentify the CT coronary angiogram as invasive as its name closely relates to a standard coronary angiogram. But, the CT coronary angiogram is a non-invasive imaging test.

### 3.3. Disease-specific protocols

Cardiac CT is frequently the first preferred imaging modality for non-invasive visualization of coronary artery stenosis (82). It primarily provides information in (a) anatomical outline, (b) functional heart assessment, (c) myocardial perfusion evaluation, (d) contrast enhancement, and (e) valvular assessment (83). See Table 5 for extensive list. As for injection protocol, they vary based on patient characteristics, injection rate, and injection duration. For example, a high injection rate is commonly combined with a high iodine concentration for coronary CTA (84).

**Table 5:** Medical condition and diagnosis that can be identified by using cardiac CT scanning.

Imaging type	Diagnosis and condition
Cardiac CT	Angina (chest pain)
	Aneurysm
	Assess for complications with procedure
	Atherosclerosis
	Blockages or narrowing
	Blood clots
	Cardiac tumor or mass
	Congenital heart disease
	Coronary artery disease
	Coronary artery stenosis
	Detects or injury to primary valves
	Dyslipidemia (abnormal cholesterol)
	Excess fluid or infection
	Heart disease
	Lipid plaque
	Obstructed coronary artery disease
	Pericarditis
	Plan for arrhythmia albation procedures
	Preparation for dissection, transcatheter/percutaneous valve procedures
	Pumping function

### 3.4. Physics and imaging sequences

The CT scanner comprises three primary systems: the gantry, computer, and operating console, and each of these main parts is composed of subcomponents (85). The gantry assembly includes all supplies related to the patient, positioning, mechanics supports, and scanner. It holds the radiation source and detectors (86). It is typically in a ring or cylinder construction, and the patient is placed inside the tube to deliver 3D images. The x-ray tube comprises two electrodes, the cathode and anode. It is vacuum-sealed and contains an electrical diode designed to emit the rays (87). The computer is specifically organized to accumulate and analyze the detector’s input to process thousands of equations simultaneously. Reconstruction rate and image quality depend on the microprocessor and internal memory (88). The third main component, the operating center, is the master control center of the CT scanner. It is used to input all the factors related to a scan. Typically, one control console is utilized by the CT operator, and the other by the physician interpreting the scans.

### 3.5. CT future

#### 3.5.1. Iterative reconstruction

**Figure 6:**
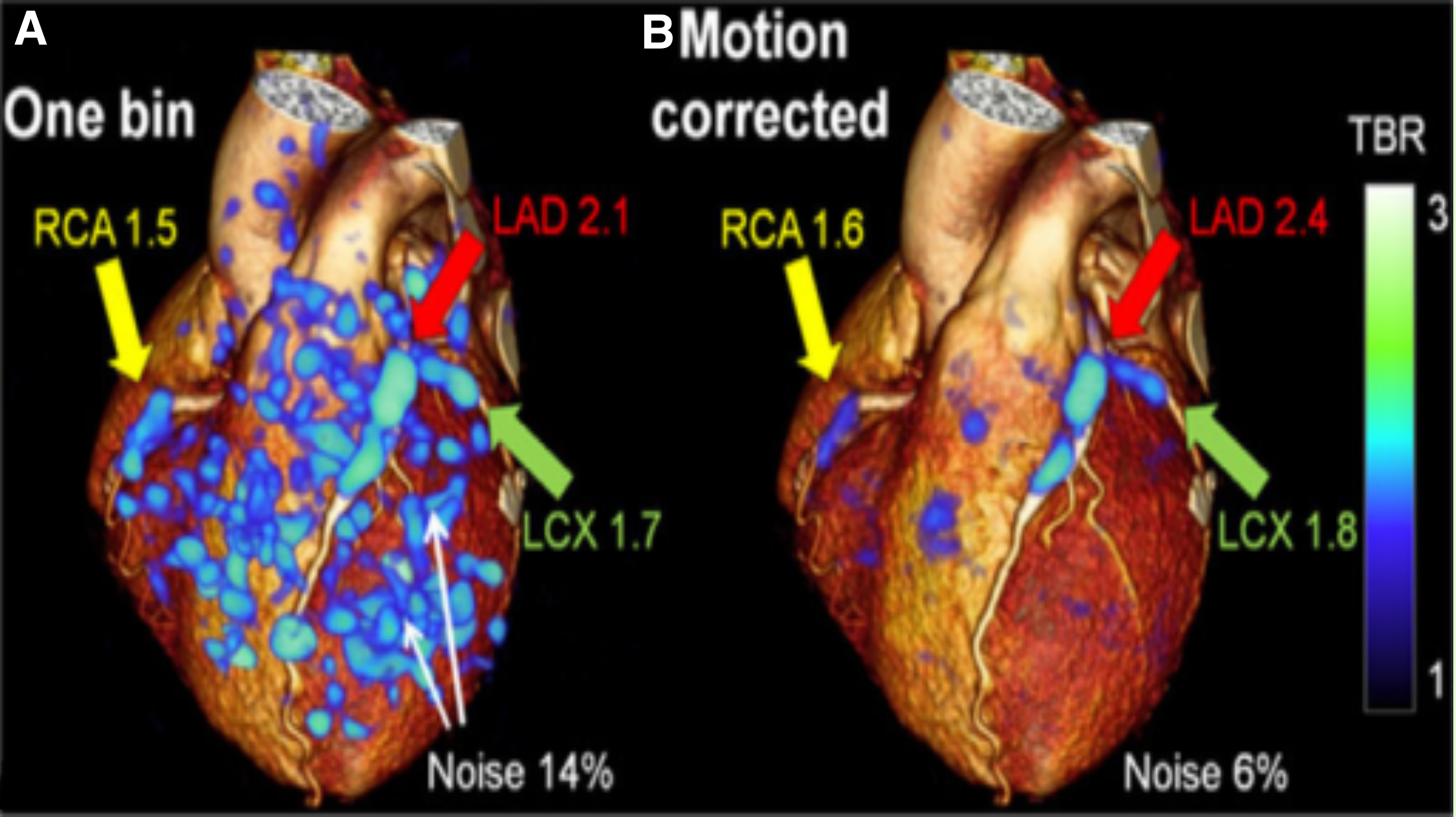
3D CT-PET: Target-to-background refinement and noise reduction with (**A**) The 1-bin image (25% of PET counts) (90); (**B**) Motion-corrected image overlayed on rendered CCTA volume. Increased plaque update is seen in RCA, LAD, and LCX coronary arteries in the high noise 1-bin image and remains clear in the motion-corrected image. RCA, right coronary artery, LAD, left anterior descending, LCX, left circumflex artery (91).

Image or iterative reconstruction (IR) is the process of generating tomographic images from x-ray projection data, or with an image assumption, then comparison to real-time measured values (89). It makes continuous adjustments via an algorithm until the two agree without increasing the radiation dose. Constant iterations work to clean the artifacts and deliver more refined images down to the pixel. An example of noise reduction and target-to-background refinement can be shown in Figure 6 below. Before IR, CT system images were created based on a filtered back projection, which was significantly slower (92). The process of IR has three separate stages. The first is the input of raw data produced by the CT scanner, and a standard filtered back-projection algorithm works to create the primary image of the heart. The second is the image reconstruction loop, a sequence of forwarding projections to create contrived raw data. The simulated data is then correlated to raw data. The image is revised, then a filtered back project is used to back-project the corrected image; this process is repeated (93). The third step is the output. A new model-based IR (MBIR) algorithm has been presented to improve image quality and lessen radiation exposure. MBIR may be advantageous as several recent studies have found MBIR to be safer and yield better outcomes (94,95).

#### 3.5.2. Multidetector CT and electron beam CT

Ordinary CT scans take 1 to 10 s per slice, but recent CT scanners have many rows of detectors, some up as high as 320 rows, that can take multiple x-rays of the heart simultaneously (96). This type of technology is called multidetector computed tomography (MDCT), and MDCT is considered the new gold standard for preoperative planning (97). In fact, the new MDCT scanners can visualize the whole heart in a ten-second breath-hold (98). Similarly, an electron beam CT (EBCT), also called an Ultrafast CT, is another innovative and fast CT scan. It takes images so quickly that it can evade the heart’s beating, a common issue with standard CT scans (99). Both MDCT and EBCT work to lower radiation emissions and speed up scan time.

#### 3.5.3. Photon-counting CT and myocardial CT perfusion

Dual-source or dual-energy CT, also known as photon-counting CT (PCCT), is an innovative technology that uses photon-counting detectors (PCDs) rather than the conventional energy integrating detectors (100). Spectral CT technology provides diverse views from a single scan instead of scanning a patient several times using various energies to focus on different tissue types. This software can also accentuate or remove chemical compounds solely based on their atomic number (e.g., iodine, calcium) (101). The PCDs count the number of incoming photons and measure this photon energy, thus resulting in a greater contrast-to-noise ratio, increased spatial resolution, multi-energy ability, and absence of electronic noise (102).

#### 3.5.4. Advanced analytics: fractional flow reserve, wall shear stress, epicardial fat enhancement

Coronary CT angiography (FFR-CT) is a logical extension of non-invasive FFR. FFR-CT uses HeartFlow Analysis to obtain 3D images of the patient’s coronary arteries, which has only previously been done with invasive procedures (103). This revolutionary HeartFlow technology utilizes advanced algorithms, artificial intelligence learning, cloud computing, computational fluid dynamics, and a team of highly trained analysts that revise the model to make it a patient-centered approach. The completed FFR-CT model is color-coded, reflecting the impact of atherosclerosis and the reduction of blood flow within the coronary arteries (104).

Similarly, wall shear stress (WSS) is an emerging notion. WSS simulation can help diagnose the significance of coronary stenosis and the probability of myocardial ischemia (105). As FFR is becoming a new gold standard for assessing the functional significance of stenosis in coronary arteries, proximal wall shear stress (WSSprox) can also help reveal plaque susceptibility (106). FFR calculates the pressure variation perpendicular to the cross-sectional area of vessels, whereas WSSprox illustrates the tangential force adjacent to the lumen walls. Kumar et al. (107) found lesions with a more elevated WSSprox were observed to show a significantly higher rate of myocardial infarction.

Epicardial fat enhancement can improve the prediction of coronary artery disease and arteriosclerosis and serve as an overall screen for cardiovascular risk (108). Deep learning methods, particularly convolutional neural networks (CNNs), have been extremely useful for cardiac image segmentation, both with MR and CT images. With the epicardial fat enhancement, some studies have found an association between higher epicardial fat and symptomatic diabetic patients, indicating clinical variables are linked to quantifiable features (109). Commandeur et al. (110) found CNN’s automation of quantification allows epicardial adipose tissue from a calcium scoring CT to be read as well as a trained radiologist typically performs. In the future, epicardial, along with subcutaneous and paracardial fat enhancement paired with CNN automation, could be utilized as a routine cardiovascular risk assessment. This would be a revolutionary proactive approach.

## 4. Artificial intelligence, machine learning, and deep learning

**Figure 7:**
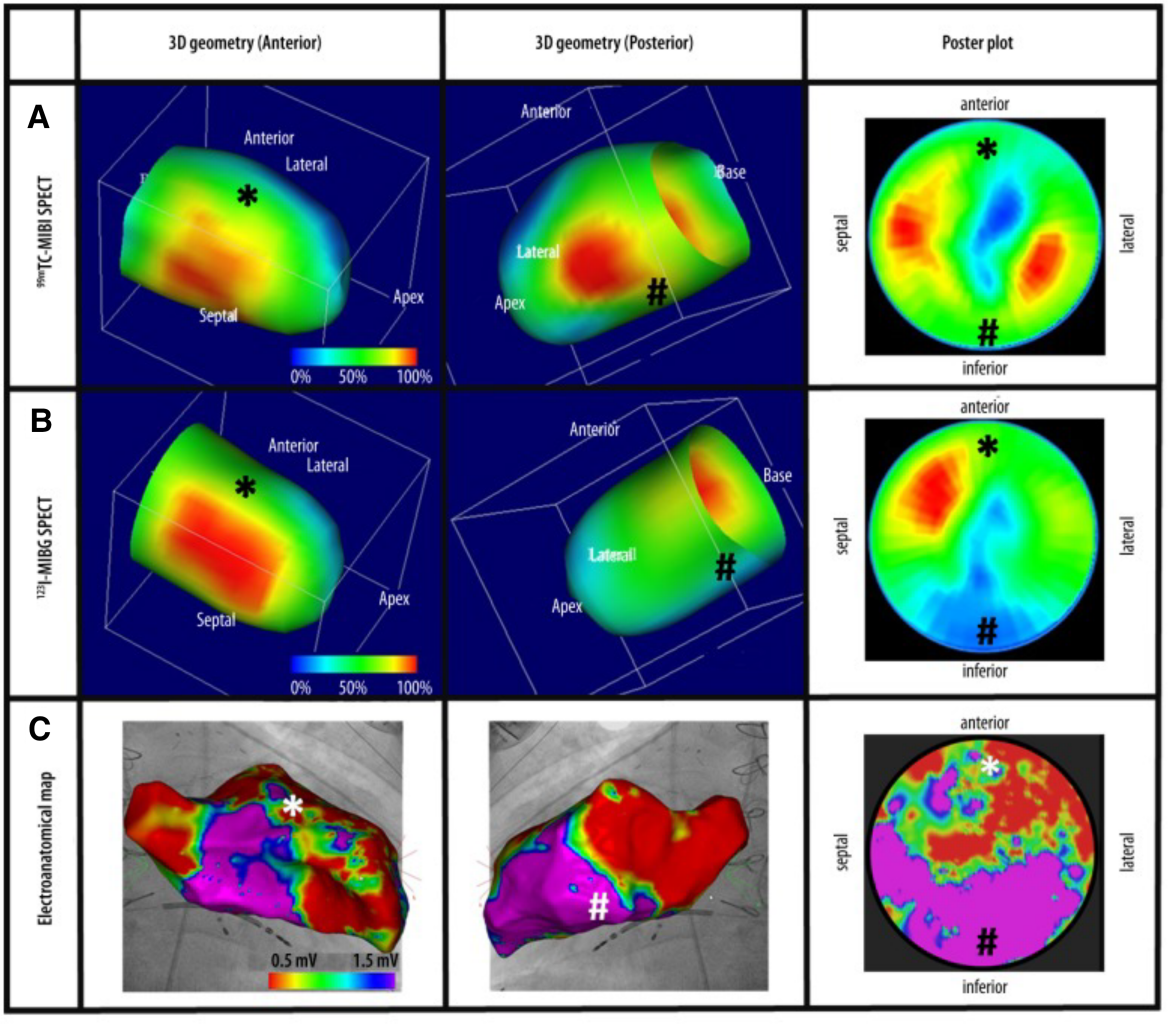
Cardiac imaging modalities combined. (**A**) Revoluntary Tc-99m-MIBI-SPECT imaging reveals anterior and inferior perfusion imaging. (**B**) I-123-MIBG-SPECT illustrates decreased sympathetic innervation at the inferior left ventricle. (**C**) Electroanatomical mapping shows extensive anterior left ventricle scarring recognized by low-voltage regions. Tc-99m-MIBI, Technetium-99m-sestamibi, I-123-MIBG, Sodium Iodide-123 Meta-Iodo-Benzyl-Guanidine (121).

There has been a surge of newly published research focused on artificial intelligence (AI), machine learning (ML), and deep learning (DL) approaches in cardiovascular imaging. AI is the concept of creating computer systems to complete tasks that typically require human intellect, such as decision-making, visual perception, and interpretation (111). For example, the Mayo Clinic uses AI to process and respond to cardiovascular scans by detecting heart disease and enriching radiology images to improve patient outcomes (112). ML is a computer system that takes AI a step further and is a subset of AI. It is a program designed to learn and adjust without direct instructions using algorithms and statistical models to study and pull deductions from patterns in data tendencies. For example, ML enables generalizability, improving disease prognostications and survival outcomes (113). One study (114) incorporated 3D ventricular systolic motion via MRI and ML. It significantly improved survival prognosis in individuals with pulmonary hypertension compared to traditional clinical, conventional imaging, hemodynamic, and functional data. Most recently, Ebrahimian and colleagues (115) found insufficient public information on validating datasets of several Food and Drug Administration (FDA)-regulated imaging-based AI/ML algorithms, recommending more objective data be published to justify clinical use. Cai and colleagues (116) found ML algorithms and ML-enabled image analysis improved the prediction, diagnosis, and classification of heart failure and hypertension, but further research is needed to investigate these cardiac conditions in terms of management. Other current limitations that exist include the lack of ML standardization to weigh all variables equally, maintain consistent quality, uphold patient safety, and ensure interoperability. DL is a subfield of machine learning that concentrates on algorithm learning techniques that utilize artificial neural network layers with representation learning, thus learning from enormous amounts of data (117). Although DL’s presumed high diagnostic and predictive value, DL tools using temporal data in image processing have not yet found their way into daily clinical practice (118). For all the potential AI, ML, and DL bring to cardiovascular imaging and improved patient outcomes, high-quality data and model assurance on unseen data sets are crucial to success (119). Ongoing improvements in algorithms can advance the standardization of imaging protocols. Parameters will also continue to advance and improve the accuracy of risk quantification (120). In the future, we anticipate AI to be integrated within the standard cardiac imaging multimodality techniques that are commonly used, see Figure 7. Moreover, AI software acan be used also in clinical reporting, automatic data analysis and computing risk scores to deliver real-time prognostication and steer patient-centered treatments.

## 5. Conclusion

Understanding the origin and narrative of cardiac imaging is essential for constructing and building more useful cutting-edge products. As reviewed in this paper, x-ray, CT, MRI, echocardiogram, and PET/SPECT have laid the foundation for cardiovascular imaging. In particular, MRI and CT have emerged as reliable, promising, and practical techniques for detecting abnormalities, diagnosing conditions, and being utilized preoperatively before procedures or corrections. Between MRI and CT, MRI has emerged as the top gold standard due to considerable advantages such as gadolinium contrast rather than harsh iodine, absence of radiation, and clear anatomical depiction of heightened resolution in 4D illustrations.

Researchers are working to optimize imaging quality, reduce scan time, lessen ionizing radiation, and improve the experience through physics, algorithms, and structural modifications for enhancement. Since the latest sophisticated imaging techniques have been developed, high amounts of “big data” are being processed into electronic health records via machine processing (e.g., through AI). As a very intriguing review stated, AI, ML, and DL are modern techniques in cardiac imaging primed to be at the forefront of a new path in precision cardiac imaging and cardiology (111).

Investigators continue to enhance image processing, reconstruction, name selection, functional assessment of coronary flow, and facilitate optimal imaging segmentation through AI. In the future, we can expect higher accuracy in detecting CAD and bypassing invasive imaging techniques, such as coronary angiography, due to objective and automated diagnosis. We predict that multimodality techniques will continue to be used in combination with AI processes. For example, AI, ML, and DL can replace manual segmentation with automation to reduce substantial noise, known as unwanted pixel values, to avoid unnecessary surgeries and misdiagnoses. Recent studies show that DL-based optimization positively impacted outcomes by noise reduction in CCTA and dynamic PET imaging (122,123). Data science and AI can be expected to continue to enhance all stages of the imaging chain with the advancement of suitable computational tools and clinical applications, which can add value to patient care.

To conclude, the goal of image optimization is not only visualization but quantification. With automation, we can increase accuracy, lessen the need for unnecessary surgeries, optimize imaging approaches for better understanding and outputs, and move towards more preventive medicine. Next, we expect development of improved algorithms to provide refined datasets that can integrate AI, ML, DL, and VR applications so the physician can visualize the heart, manipulate it, and slice it for dissection to determine diagnosis due to additional AI, ML, and DL-integrated tools.
